# Genetic Association Study of IL2RA, IFIH1, and CTLA-4 Polymorphisms With Autoimmune Thyroid Diseases and Type 1 Diabetes

**DOI:** 10.3389/fped.2020.00481

**Published:** 2020-08-21

**Authors:** Hanna Borysewicz-Sańczyk, Beata Sawicka, Natalia Wawrusiewicz-Kurylonek, Barbara Głowińska-Olszewska, Anna Kadłubiska, Joanna Gościk, Agnieszka Szadkowska, Aleksandra Łosiewicz, Wojciech Młynarski, Adam Kretowski, Artur Bossowski

**Affiliations:** ^1^Department of Pediatrics, Endocrinology, Diabetology With Cardiology Division, Medical University of Bialystok, Bialystok, Poland; ^2^Department of Endocrinology and Diabetes With Internal Medicine, Medical University of Bialystok, Bialystok, Poland; ^3^Faculty of Computer Science, University of Technology, Bialystok, Poland; ^4^Department of Pediatrics, Diabetology, Endocrinology and Nephrology, Medical University of Lodz, Lodz, Poland; ^5^Department of Pediatrics, Oncology, Hematology and Diabetology, Medical University of Lodz, Lodz, Poland; ^6^Department of Endocrinology and Diabetes With Internal Medicine, Medical University in Bialystok, Bialystok, Poland

**Keywords:** Graves' disease (GD), Hashimoto's thyroiditis (HT), type 1 diabetes (T1D), genetic susceptibility, single nucleotide polymorphism (SNP), IL2RA, IFIH1, CTLA-4

## Abstract

Autoimmune thyroid diseases (AITDs) which include Graves' disease (GD) and Hashimoto's thyroiditis (HT) as well as type 1 diabetes (T1D) are common autoimmune disorders in children. Many genes are involved in the modulation of the immune system and their polymorphisms might predispose to autoimmune diseases development. According to the literature genes encoding IL2RA (alpha subunit of Interleukin 2 receptor), IFIH1 (Interferon induced with helicase C domain 1) and CTLA-4 (cytotoxic T cell antigen 4) might be associated with autoimmune diseases pathogenesis. The aim of the study was to assess the association of chosen single nucleotide polymorphisms (SNPs) of IL2RA, IFIH1, and CTLA-4 genes in the group of Polish children with AITDs and in children with T1D. We analyzed single nucleotide polymorphisms (SNPs) in the IL2RA region (rs7093069), IFIH1 region (rs1990760) and CTLA-4 region (rs231775) in group of Polish children and adolescents with type 1 diabetes (*n* = 194) and autoimmune thyroid diseases (GD *n* = 170, HT *n* = 81) and healthy age and sex matched controls for comparison (*n* = 110). There were significant differences observed between T1D patients and control group in alleles of IL2RA (rs7093069 T > C) and CTLA-4 (rs231775 G > A). In addition, the study revealed T/T genotype at the IL2RA locus (rs7093069) and G/G genotype at the CTLA-4 locus (rs231775) to be statistically significant more frequent in children with T1D. Moreover, genotypes C/T and T/T at the IFIH1 locus (rs1990760) were significantly more frequent in patients with T1D than in controls. We observed no significant differences between AITD patients and a control group in analyzed SNPs. In conclusion, we detected that each allele T of rs7093069 SNP at the IL2RA locus and G allele of rs231775 SNP at the CTLA-4 locus as well as C/T and T/T genotypes of rs1990760 SNP at the IFIH1 locus are predisposing in terms of T1D development. Thereby, we confirmed that IL2RA, IFIH1, and CTLA-4 gene locus have a role in T1D susceptibility. The analysis of selected SNPs revealed no association with AITDs in a group of Polish children and adolescents.

## Introduction

The underlying cause of autoimmune diseases is the loss of immune tolerance to tissue-specific antigenic peptides which leads to immune response directed against one's own body's cells. Still not completely understood, complex immune mechanisms including the dysfunction of the immune system might be involved in the autoimmune diseases pathogenesis (1). Among the most common chronic autoimmune endocrine disorders in children there are autoimmune thyroid diseases (AITDs) which include Graves' disease (GD) and Hashimoto's thyroiditis (HT) as well as type 1 diabetes (T1D) (2). In children with autoimmune thyroiditis immune reactions are directed against the cells of thyroid gland. In GD the thyrotropin receptor (TSH-R) is activated with antibodies causing the overactivity of the thyroid gland, while in HT humoral and cell-mediated thyroid injury leads to destruction of thyroid cells and hypothyroidism as a consequence (3). In diabetic patients an inappropriate immune reaction results in autoreactive T-cell infiltration and production of tissue specific autoantibodies which cause the destruction and dysfunction of the insulin secreting pancreatic beta cells and insulin deficiency (4). The mechanisms leading to development of these diseases remain unknown, however numerous data indicate that apart from the environmental factors there is a strong genetic susceptibility to the autoimmune diseases (5–8). The relevance of genetic factors is evident from clustering of AITDs or T1D within families, in particular monozygotic and dizygotic twins (9, 10). Many genes might be involved in the modulation of the immune system and some of them were recently found to influence autoimmune endocrine disorders development. Moreover, recent studies have demonstrated that some genetic risk factors for autoimmunity are shared between diseases, contributing to the development of more than one autoimmune disorder (10). Current publications showed association between autoimmune diseases and chromosome 10p15 region for IL2RA (interleukin 2 receptor-α), chromosome 2q33 region for CTLA-4 (cytotoxic T-lymphocyte antigen-4) and chromosome 2q24 region for IFIH1 (interferon induced with helicase C domain 1) (11, 12). The most frequent type of human genome variation are single nucleotide polymorphisms (SNPs) providing powerful tools for a variety of medical genetic studies (13). Although certain polymorphic variants of genes encoding IL2AR, CTLA-4, or IFIH1 have been reported to implicate T1D and ATDs development in adults, there are only few studies focusing on children (14–18).

Interleukin 2 (IL2) is a lymphocytes growth factor playing an important role in modulation of immune homeostasis as an essential self-tolerance regulator (19, 20). Its action is mediated by a quaternary receptor signaling complex (IL2R) containing α, β and a common γ chain receptors (21, 22). Alpha subunit of the IL2 receptor, IL2Rα (also known as CD25), encoded by the interleukin 2 receptor α gene (IL2RA), plays a key role in mediating interleukin 2 immunoregulatory function. The expression of IL2RA has been described at high levels on the surface of the regulatory T cells (Tregs), a population of T cells with an ability to inhibit autoreactive T cells (23). Further studies indicated IL2RA's essential role in sensitizing T cells for induced cell death (22) that is crucial for their function as a suppressor for T cell immune responses to auto-, alloantigens, as well as tumor antigens and antigens deriving from pathogens (24). SNPs of genes influencing Treg function, such as IL2RA, may cause an increased risk of autoimmune disease.

Interferon induced with helicase C domain 1 (IFIH1) also known as Helicard or melanoma differentiation-associated gene 5 (MDA-5), plays an essential role in body immune reactions against viruses. IFIH1 belongs to the family of RNA helicases binding viral RNA (25, 26). IFIH1 protein acts as a detector of viral double strand RNA (dsRNA) and causes the apoptosis of virally infected cells (27). The studies suggest that variants of genes involved in the inflammation responses might have the potential to alter their function and expression (28). Previously established relationships between autoimmune diseases development and viral infection might have a molecular basis provided by genetic variants of IFIH1 (29). The SNPs of IFIH1 could cause the abnormal activation of antiviral defenses signaling leading to the autoimmune disease development.

Cytotoxic T-lymphocyte antigen-4 (CTLA-4), also known as CD152, encodes T cell receptors responsible for the attenuation of immune response. CTLA-4 acts by delivering an inhibitory signal decreasing cytokine production, activation and proliferation of T lymphocytes (30–32). Polymorphic variants of CTLA-4 gene are implicated in dysregulation of immune homeostasis due to an aberrant activation of T-lymphocytes in the periphery which may cause the infiltration of glands leading to their dysfunction and autoimmune disease development. According to the literature, common CTLA-4 polymorphisms have been found to confer susceptibility to T1D, AITDs (12, 33) and other autoimmune disorders (34, 35).

Since it has been suggested that multiple genes are associated with pathogenesis of autoimmune disorders and some autoimmune diseases might share the same genetic background of co-occurrence within individuals and families, the aim of the study was to assess the association of chosen single nucleotide polymorphisms of IL2RA, IFIH1, and CTLA-4 genes in the group of Polish children with AITDs and in children with T1D. In our study we hypothesized that the same polymorphisms of IL2RA, IFIH1, or CTLA-4 genes might be associated with AITDs and might predispose to T1D development.

## Materials and Methods

**Figure 1:**
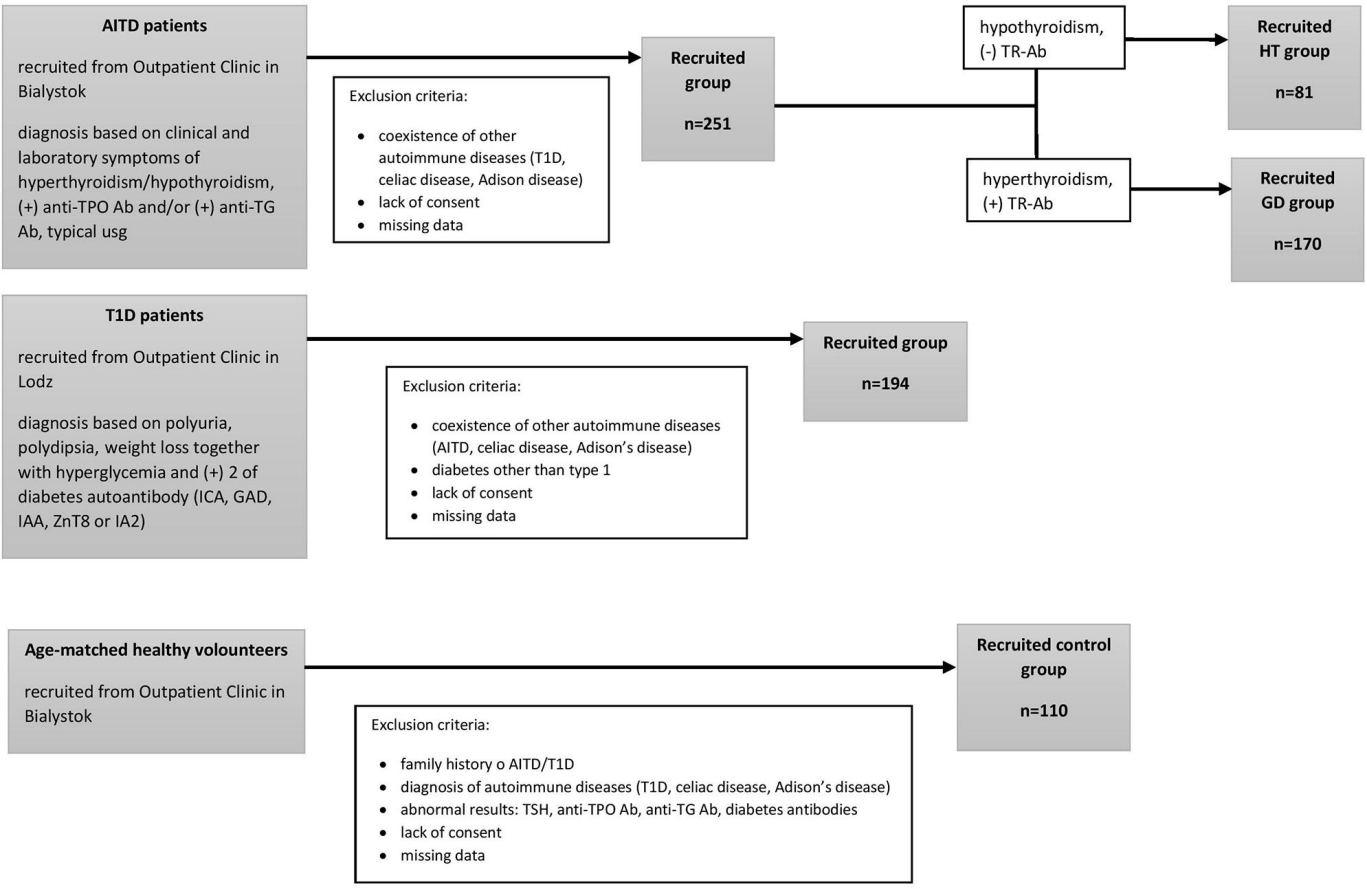
The study selection process.

We performed this original research study in the group of 81 HT patients (mean age, 15.2 ± 2.2 years) 170 GD patients (mean age, 16.5 ± 2 years) recruited from the Outpatient Clinic in Bialystok and 194 patients with T1D (mean age, 10.18 ± 3.4 years) recruited from the Outpatient Clinic in Lodz. None of the patients suffered from more than one of these conditions. The qualifying criteria for patients and controls are presented in Figure 1. AITDs were diagnosed according to the Polish Endocrinology Association guidelines which correspond with the guidelines of the European Society for Pediatric Endocrinology. The inclusion criteria for patients with AITDs were based on medical history, physical examination, laboratory and ultrasound investigations. GD was diagnosed in children with large goiter, hyperthyroidism in laboratory tests and positive thyrotropin receptor antibodies (TR-Ab). HT patients developed clinical and biochemical symptoms of hypothyroidism and demonstrated presence of anti-TPO and/or anti-TG autoantibodies. T1D was diagnosed according to the Polish Diabetes Association guidelines which correspond with the guidelines of the WHO and was based on clinical symptoms, hyperglycemia, low fasting C-peptide levels and the presence of diabetes autoantibodies (islet cell antibodies - ICA, glutamic acid decarboxylase antibodies – GAD, insulin autoantibodies – IAA, zinc transporter 8 autoantibodies - ZnT8 or antibodies to protein tyrosine phosphatase - IA2). The control group consisted of 110 healthy volunteers (mean age, 16.3 ± 3 years). All controls had no history of HT, GD or T1D, were euthyroid and had no thyroid and diabetes autoantibodies. For the treatment patients with GD received methimazole at a dose of 0.3–1.0 mg/kg/d together with propranolol (0.5–1.0 mg/kg/d) orally. HT patients were treated with L-thyroxine (1 mcg/kg/d) orally. Diabetic patients were receiving insulin in appropriate doses. Before enrolment, all parents of patients and controls and all children over 16 years old gave informed consent. The study protocol was accepted by the Local Ethical Committee at the Medical University of Bialystok and adheres to the Declaration of Helsinki. Additional information regarding the study subjects are shown in Table 1.

**Table 1:** Clinical characteristics of patients with T1D, ATD and controls.

	**T1D (mean ± SD)**	p^*^	**HT (mean ± SD)**	p^**^	**GD (mean ± SD)**	p^***^	**Controls (mean ± SD)**
n (F/M)	194 (88/106)		81 (59/22)		170 (126/44)		110 (50/60)
Age (years)	10.18 ± 3.9	<0.01	15.2 ± 2.2	NS	16.5 ± 2	NS	16.3 ± 3
Weight (kg)	43.4 ± 17.0	<0.01	58 ± 5.28	NS	55.19 ± 2.39	NS	60.9 ± 7.8
Height (cm)	148.5 ± 17.8	<0.01	154.26 ± 4.14	NS	162.19 ± 2.69	NS	160 ± 8
BMI (kg/m^2^)	19.0 ± 3.9	<0.01	24.45 ± 1.33	NS	21.1 ± 2.1	0.012	23.78 ± 2.5
HBA1c	11.9 ± 2.9		-		-		-
TSH (mIU/l)	2.19 ± 1.57	NS	9.87 ± 4.37	<0.025	0.37 ± 0.1	<0.01	3.04 ± 0.72
fT4 (ng/dl)	-		1.21 ± 0.03	NS	3.6 ± 1.4	<0.001	1.1 ± 0.17
fT3 (pg/ml)	-		3.08 ± 0.5	NS	7.19 ± 1.65	<0.001	3.79 ± 0.18
TR-Ab (IU/l)^a^	-		0.5 ± 0.32	NS	11.56 ± 2.11	<0.001	0.4 ± 0.2
Anti-TPO Ab (IU/ml)	-		329.91 ± 92.93	<0.001	331.97 ± 58.12	<0.001	26.72 ± 6.8
Anti-TG Ab (IU/ml)	-		620.98 ± 240.34	<0.001	347.49 ± 86.7	<0.001	41.64 ± 12.1
ICA	65% (+)		-		-		-
GAD	79% (+)		-		-		-
IA2	67% (+)		-		-		-
ZnT8	63% (+)		-		-		-
IAA	43% (+)		-		-		-
Treatment	Insulin		L-thyroxine		Methimazole		-

a *TR-Ab were analyzed in selected group of patients with HT (n = 43)*.

* *Statistical significance between patients with T1D and controls*.

** *Statistical significance between patients with HT and controls*.

*** *Statistical significance between patients with GD and controls*.

Blood for analysis was collected in the morning from the basilic vein. Serum levels of thyrotropin (TSH), free thyroxine (fT4), and free triiodothyronine (fT3) were evaluated on electrochemiluminescence “ECLIA” with Cobas E411 analyzer (Roche Diagnostics). Ranges for TSH were: 0.28–4.3 (μIU/l), for fT4: 1.1–1.7 ng/dl and for fT3: 2.3–5.0 pg/ml. Antibodies against TSH-Receptor (TR-Ab), Thyroid Peroxidase (TPO) and Thyroglobulin (TG) were determined using ECLIA with Modular Analytics E170 analyzer (Roche Diagnostics). The positive values were: > 1.75 U/l for TR-Ab, > 34 IU/mL for anti-TPO-Ab and > 115 IU/mL for anti-TG-Ab. The conventional anti-diabetes autoantibodies were detected in serum samples: ICA with immunofluorescence, GAD, IA2, and ZnT8 by ELISA (RSR, USA) and IAA with RIA (CisBiointernational, France and RSR, USA). The positive values for ICA, GADA, IA2 and IAA positivity were 10 Juvenile Diabetes Foundation units, 10 U/ml and 20 U/ml and 7%/0.4 U/ml respectively.

The DNA was extracted with a classical salting-out method from the blood leukocytes. All study subjects were genotyped for SNPs at three loci: IL2RA (rs7093069), IFIH1 (rs1990760), and CTLA-4 (rs231775). TaqMan SNP genotyping assay (Applied Biosystems, Foster City, CA) was used for all genotyping. For this, polymorphisms fluorogenic TaqMan probes were used. Reactions were performed in a 7900HT fast real-time PCR system (Applied Biosystems) according to the conditions: 10 min at 95 C for starting AmpliTaq Gold activity, 40 cycles of 95 C for 15 s and 60 C for 1 min. A sample without template served as a negative control and it was helpful to detect any false positive signal caused by contamination. All SNPs were analyzed in duplicates.

Median unbiased estimator (mid-p) of odds ratio, the exact confidence interval and associated *p*-value obtained with the mid-p method were used to determine any association between genotype or allele occurrence and patient's status (36). Either parametric or non-parametric methods, according to the normality and homogeneity of variance assumptions, were used to assess whether there are statistically significant differences between groups defined by genotypes and quantitative features. False discovery rate *p*-value adjustment method was applied due to the issue of multiple testing during the *post-hoc* analysis (37). As proposed in (38) measure D' of linkage disequilibrium was used. For all calculations *P*-value of < 0.05 was considered to be significant. The R software (Vienna, Austria) environment was exploited for all calculations (39). Statistical power calculation with respect to the total sample size was calculated with the use of G^*^Power ver. 3.1.9.6 software (40). Cohen's w was applied as a measure of effect size. Hardy-Weinberg Equilibrium was checked with the utilities of the genetics package (41).

## Results

**Figure 2:**
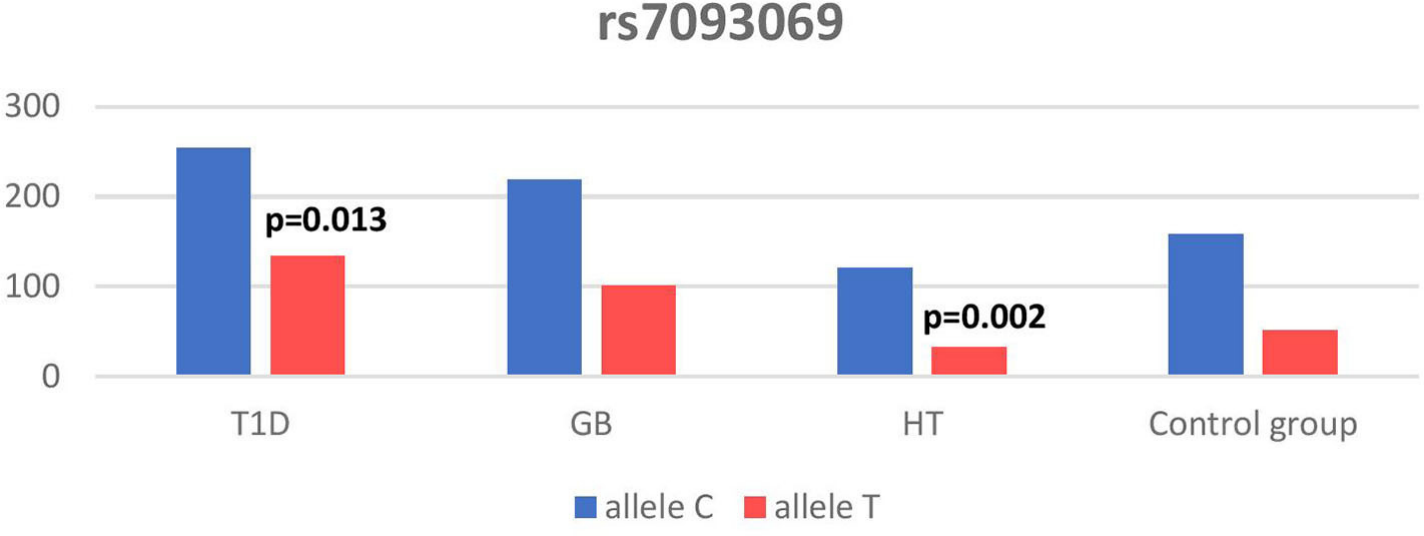
Number of alleles in rs7093069 polymorphism (n) in the IL2RA region.

**Figure 3:**
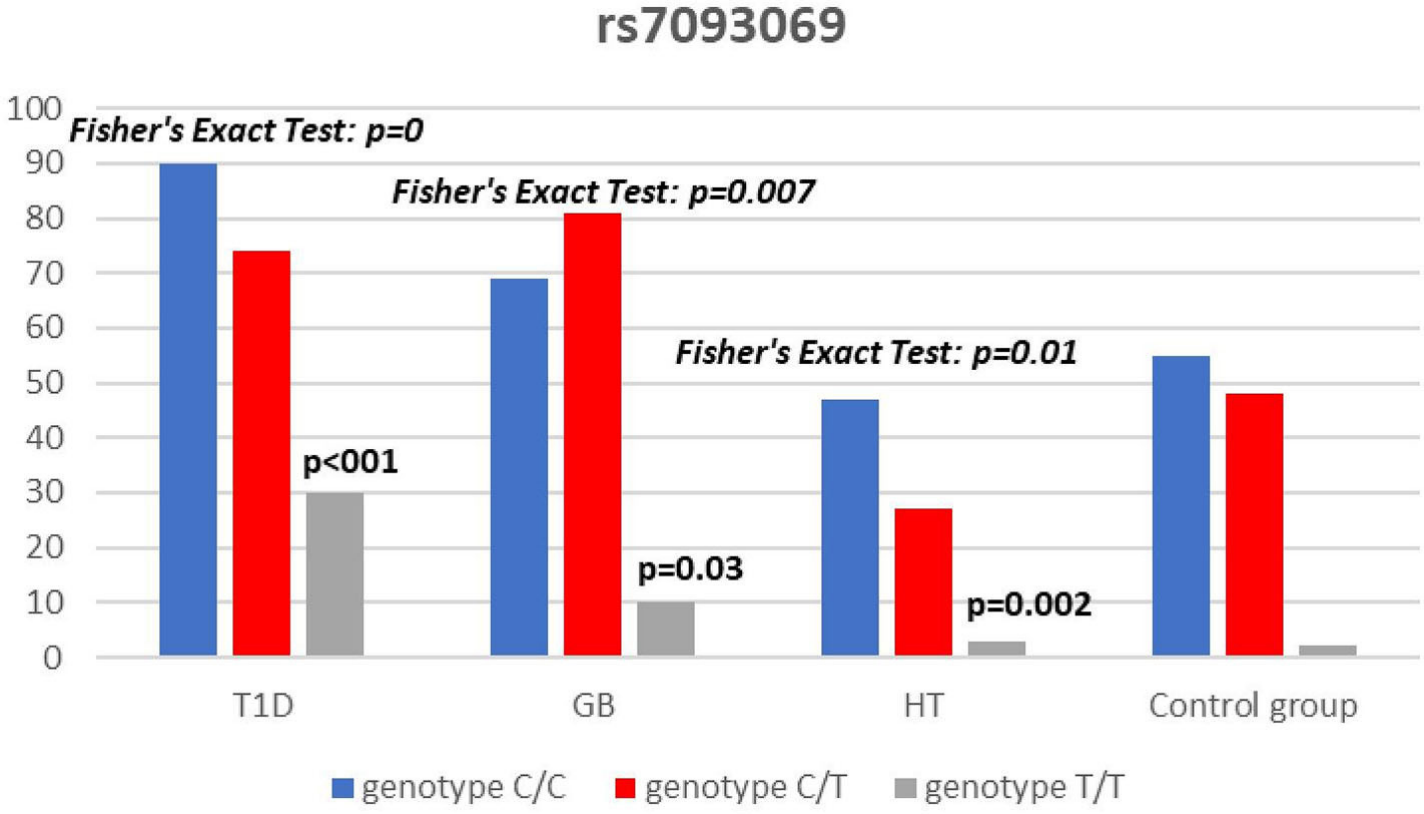
Number of genotypes in rs7093069 polymorphism (n) in the IL2RA region.

**Figure 4:**
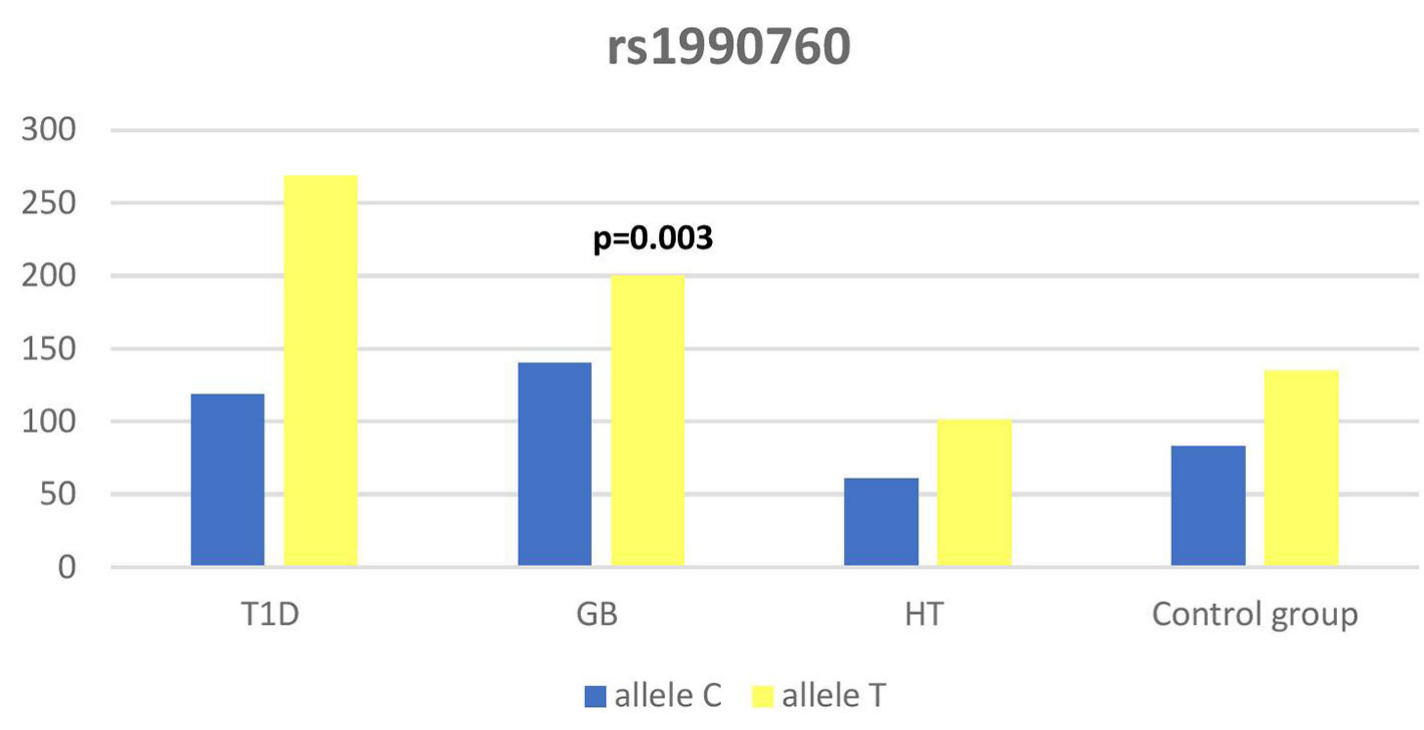
Number of alleles in rs1990760 polymorphism (n) in the IFIH1 region.

**Figure 5:**
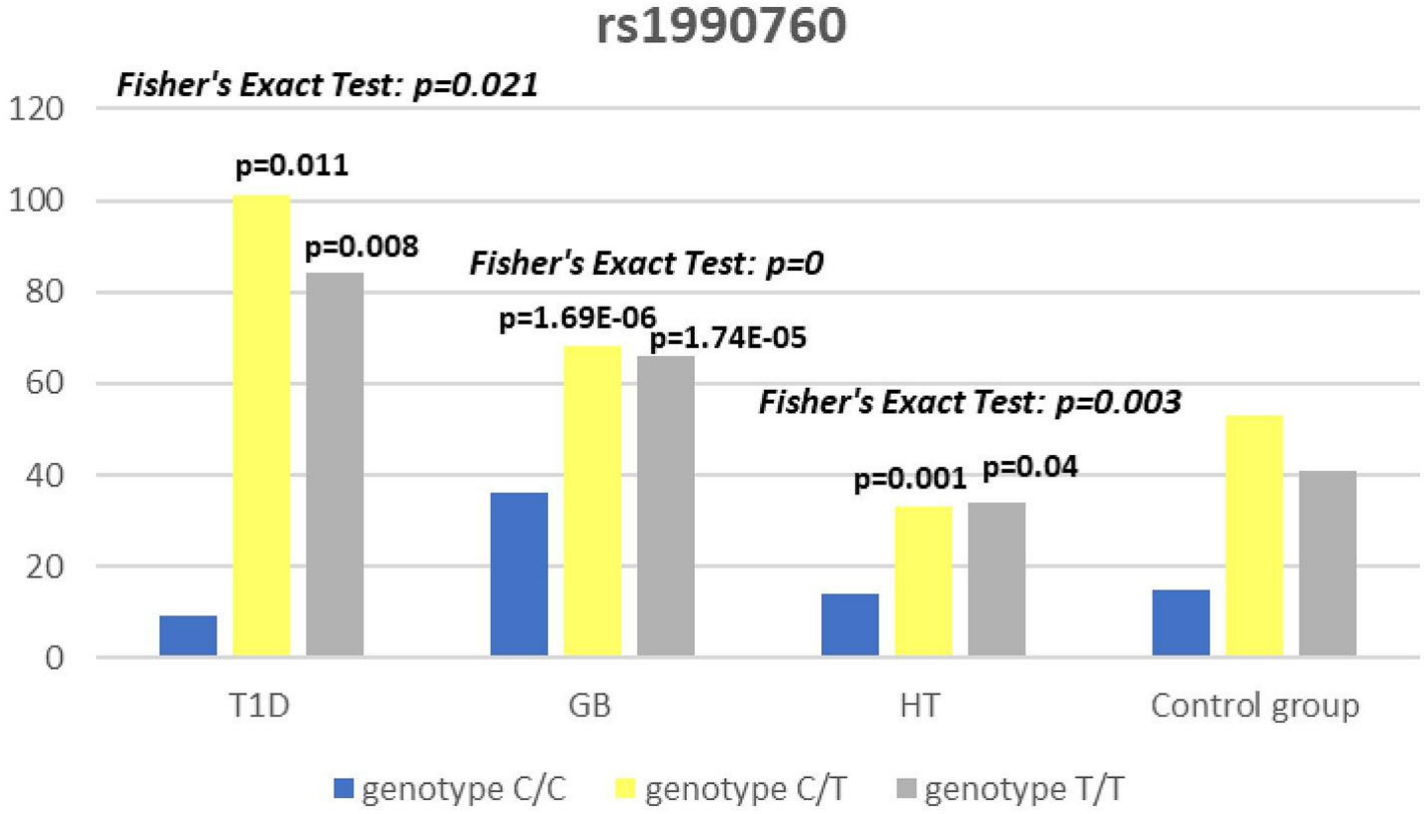
Number of genotypes in rs1990760 polymorphism (n) in the IFIH1 region.

**Figure 6:**
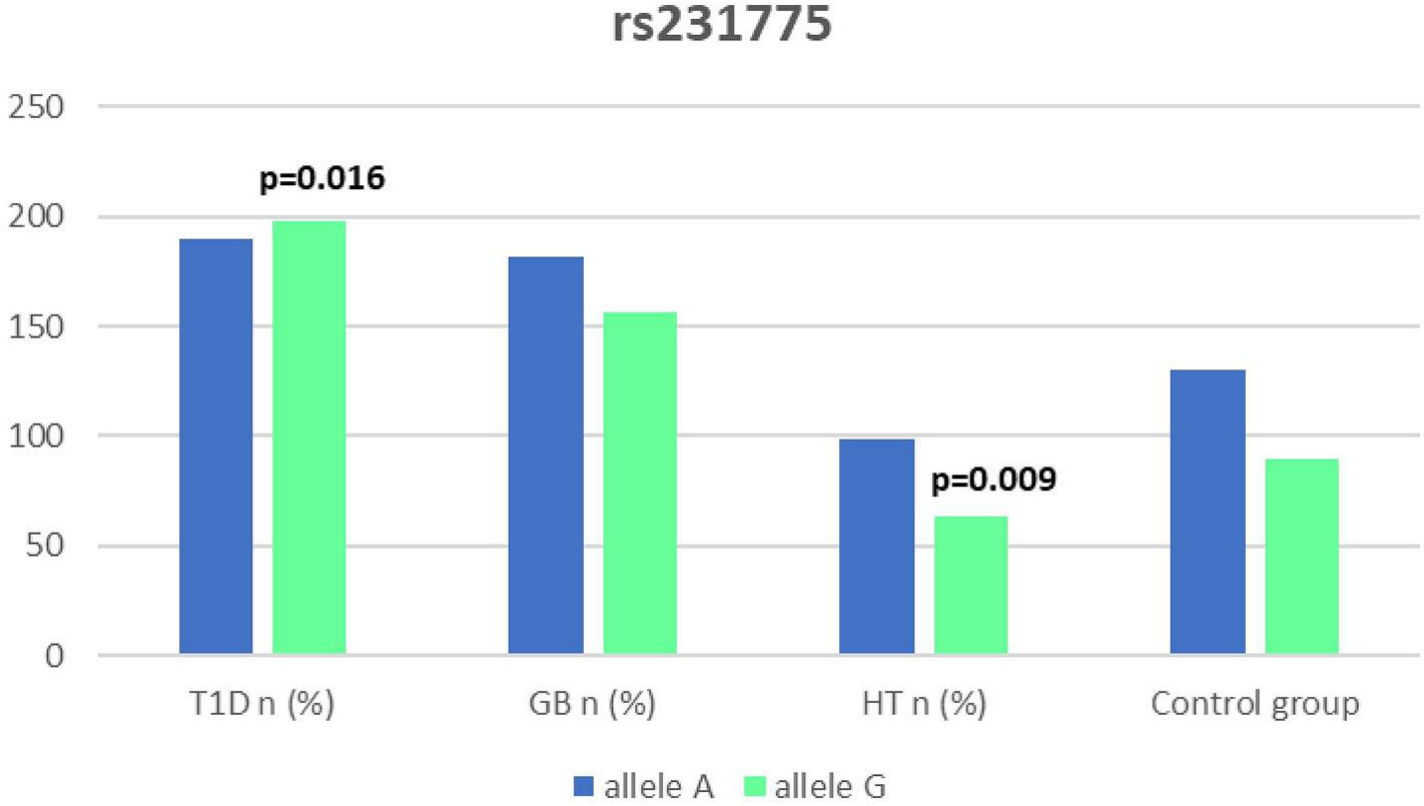
Number of alleles in rs7231775 polymorphism (n) in the CTLA-4 region.

**Figure 7:**
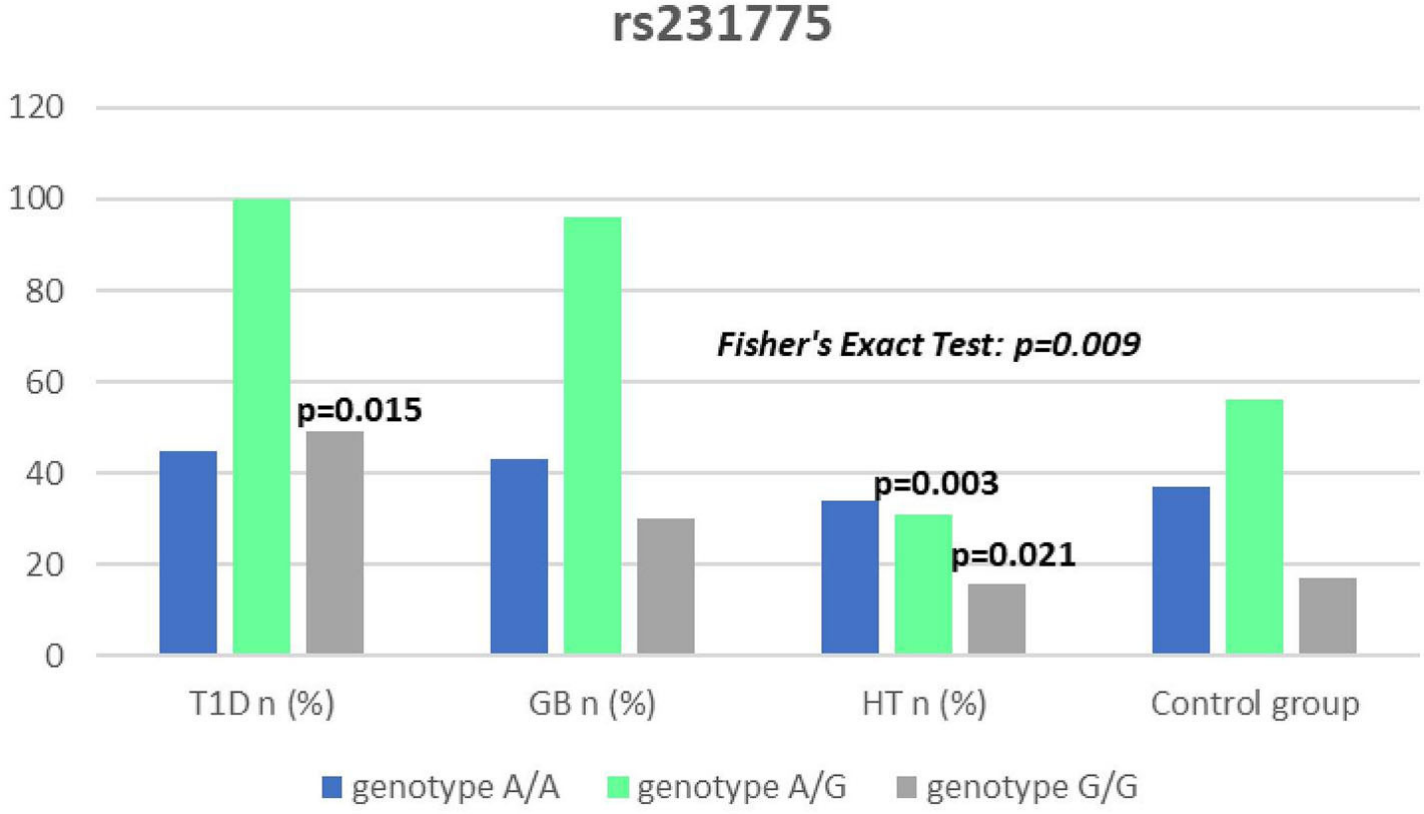
Number of genotypes in rs321775 polymorphism (n) in the CTLA-4 region.

Significant associations were observed between T1D patients and controls in alleles of IL2RA (rs7093069 T > C, *P* = 0.013, OR = 1.59, 95%CI = 1.10–2.34, the power of the test was 0.999, estimated effect size w was equal to 0.231, with the total sample size *n* = 598) (Figure 2) and CTLA-4 (rs231775 G > A, *P* = 0.016, OR = 1.50, 95%CI = 1.07–2.10, the power of the test was 0.9935, estimated effect size w was equal to 0.203, with the total sample size *n* = 608) (**Figure 6**). In addition the study revealed T/T genotype at the IL2RA locus (rs7093069) and G/G genotype at the CTLA-4 locus (rs231775) to be statistically significantly more frequent in children with T1D (*P* < 0.001, OR = 8.50, 95%CI = 2.42–58.38, the power of the test 1, estimated effect size w equal to 1.000, with the total sample size *n* = 299 and *P* = 0.015, OR = 2.34, 95%CI = 1.17–4.84, the power of the test was 0.997, estimated effect size w was equal to 0.320, with the total sample size *n* = 304 respectively) (Figures 3, **7**). Moreover, genotypes C/T and T/T at the IFIH1 locus (rs1990760) were significantly more frequent in patients with T1D than in controls (*P* = 0.011, OR = 3.13, 95%CI = 1.29–8.00 and *P* = 0.008, OR = 3.36, 95%CI = 1.36–8.73 respectively, the power of the test 0.918, estimated effect size w was equal to 0.240, with the total sample size *n* = 303) (**Figure 5**). In contrast there were no significant differences between AITD patients and control group in analyzed SNPs. Further analysis revealed statistically significant differences between GD and T1D patients: T/T genotype at the IL2RA locus (rs7093069) was more frequent in T1D patients (*P* = 0.03, OR = 2.27, 95%CI = 1.06–5.22, the power of the test 1, estimated effect size w equal to 0.449, with the total sample size *n* = 354) (Figure 3), as well as T alleles, C/T and T/T genotypes at the IFIH1 locus (rs1990760) were more often in diabetic patients (*P* = 0.003, OR = 1.58, 95%CI = 1.16–2.14, the power of the test 0.998, estimated effect size w equal to 0.203, with the total sample size *n* = 728, *P* = 1.69E-06, OR = 5.82, 95%CI = 2.72–13.69 and *P* = 1.74E-05, OR = 4.99, 95%CI = 2.32–11.80, the power of the test 0.999, estimated effect size w equal to 0.402, with the total sample size *n* = 364, respectively) (Figures 4, 5). Comparing HT children with T1D patients T allele and T/T genotype at the IL2RA locus (rs7093069) were statistically significant more frequent in patients with diabetes (*P* = 0.002, OR = 1.92, 95%CI = 1.25–3.02, the power of the test 0.999, estimated effect size w equal to 0.314, with the total sample size *n* = 558 and *P* = 0.002, OR = 4,96, 95%CI = 1.64–22.39, the power of the test 1, estimated effect size w equal to 0.449, with the total sample size *n* = 354, respectively) (Figures 2, 3). C/T and T/T genotypes at IFIH1 locus (rs1990760) were more frequent in T1D patients than in HT children (*P* = 0.001, OR = 4.67, 95%CI = 1.86–12.33, and *P* = 0.004, OR = 3.78, 95%CI = 1.50–9.99 respectively, the power of the test 0.990, estimated effect size w equal to 0.309, with the total sample size *n* = 282) (Figure 5). G alleles, A/G and G/G genotypes at the CTLA-4 locus (rs231775) were more frequent in T1D than in HT group (*P* = 0.009, OR = 1.63, 95%CI = 1.12–2.38, the power of the test 0.999, estimated effect size w equal to 0.246, with the total sample size *n* = 566, *P* = 0.003, OR = 2.42, 95%CI = 1.32–4.45 and *P* = 0.02, OR = 2.29, 95%CI = 1.12–4.81 the power of the test 0.999, estimated effect size w equal to 0.385, with the total sample size *n* = 283, respectively) (Figures 6, 7). There were no violations detected according to Hardy-Weinberg Equilibrium: *p* = 0.7369 for rs1990760, *p* = 0.6331 for rs231775 and *p* = 0.3001 for rs7093069.

## Discussion

Variants of IL2RA gene had been recently associated with susceptibility to several autoimmune diseases such as T1D (42), AITDs (16, 43), rheumatoid arthritis (44) or juvenile idiopathic arthritis (45) that implies the possible general effect on predisposition to autoimmunity of this region.

In our study T allele at the rs7093069 locus of the Il2RA gene was observed to be more frequent in diabetic patients compared to healthy children and HT individuals. Moreover, T/T genotype of analyzed IL2RA SNP was more frequent in T1D patients compared to healthy children as well as AITD children, indicating strong susceptibility of T allele and T/T genotype to diabetes development.

In the literature there are numerous reports demonstrating the association of SNPs in the IL2RA gene with T1D but none of them alone explains the predisposition to the disease. In the meta-analysis of Tang et al. authors analyzed the results of 10 independent studies of polymorphisms in the IL2RA gene. They confirmed rs11594656, rs2104286, and rs41295061 SNPs to be the most associated risk factors for T1D development (46), but the analysis did not investigate rs7093069 locus we analyzed in our study. Further studies of Qu et al. showed two other SNPs in the IL2RA gene that were significantly associated with T1D - rs706778 and rs3118470 (47). Moreover, Kawasaki et al. found in their study SNPs rs706778 and rs3118470 of the IL2RA in patients with T1D to be associated with acute-onset of the disease (24). According to the study of Lowe et al. ss52580101 locus in the IL2RA gene is most associated with T1D, however the authors emphasized the importance of other locus of this region (48). In addition they indicated that SNPs in IL2RA locus associated with T1D influence the soluble form of IL2RA concentration. Klinker et al. demonstrated the association of SNPs in the IL2RA locus to be an important determinant of age at diagnosis time in a group of Finnish type 1 diabetes subjects (49). The study of Fichna et al. in a group of 445 Polish T1D patients and 671 healthy control subjects confirmed the association of Il2RA single nucleotide polymorphism rs11594656 and rs3118470 but not for rs7093069, that was on the contrary to our study (50). In another work of the authors they indicate the association of other polymorphism (rs6822844) with the disease (51).

In our work, rs7093069 polymorphisms in the IL2RA gene did not show any correlation with AITDs in the population of Polish children. However, Chistiakov et al. examined other SNPs in the region and revealed the allele A of rs41295061 SNP to be significantly associated with increased risk of GD in a group of 1474 Russian patients (44). Furthermore, the study revealed that patients carrying two copies of the haploid genotype AA/AA had elevated levels of serum IL2Rα in both GD patients and healthy controls. Brand et al. found an association between GD and healthy control for the ILR2A region for 20 SNPs (among others rs7093069). The pattern of association was similar to that found in T1D patients (16). In our study there was no significant difference between AITD patients and controls in analyzed rs7093069 SNPs but we revealed statistically significant differences between GD and T1D patients in that locus, as T/T genotype was more frequent in T1D patients.

The IFIH1 gene locus has been recently defined as a candidate for susceptibility to autoimmune diseases like vitiligo, T1D and GD (14, 17, 52). Our current study confirms that the rs1990760 in IFIH1 gene is substantially associated with T1D as we found that genotypes C/T and T/T at the IFIH1 locus (rs1990760) is significantly more frequent in patients with T1D than in controls. The genetic predisposition of rs1990760 (A946T) SNP toT1D was first reported in a GWA (53). The data were recently confirmed by the other investigators in the multipopulation analyses (54, 55). In the comprehensive meta-analysis by Jermendy et al. revealed that polymorphism in rs1990760 was associated with T1D in both Finnish population (with the high-incidence of the disease) and Hungarian population (with medium-incidence of the disease) and G allele (vs A allele) significantly decreased the risk of T1D (56). Furthermore, in the meta-analysis Cen and colleagues analyzed 19 studies and revealed that the IFIH1 rs1990760 T allele influences susceptibility to T1D and other autoimmune diseases like systemic lupus erythematosus, multiple sclerosis and rheumatoid arthritis (57). In a group of Polish adult individuals the association of allele A in rs1990760 locus with susceptibility to T1D was confirmed and the cumulative effect of other polymorphic variants in IFIH1 was observed (58). Genetic association between IFIH1 polymorphism and T1D development might be explained by the link of the disease with prior viral infection. Moreover, Jermedy et al. found in their work that there is a seasonal manifestation of T1D was related to rs1990760 polymorphism as the AA genotype, that predisposes to the disease, was more frequent in patients who developed the disease in summer than in patients with onset in winter indicating that this virus receptor gene might influence T1D manifestation mainly during the summer months (59).

Present work did not reveal any significant differences in allele or genotype frequencies for rs1990760 polymorphism between GD and HT patients in comparison to healthy control subjects in Polish population. In our study T alleles, C/T and T/T genotypes at the IFIH1 locus (rs1990760) were more often in diabetic patients when compared with GD and HT patients. The association with GD and polymorphism rs1990760 (A946T) in IFIH1 gene was observed by Sutherland et al. in the study involving the United Kingdom population (17). In a previous analysis of Polish 142 pediatric patients with AITDs T alleles of rs1990760 were associated with GD in mails. Similarly in HT patients, rs1990760 T alleles were more frequent in males in comparison to healthy subjects (52). In contrast there were no significant differences for rs1990760 IFIH1 polymorphism in German patients with GD and HT in comparison to healthy control in the study of Penna-Martinez et al. (60). Similarly Zhao et al. observed no significant differences in the allele and genotype frequencies for this polymorphism between GD patients and healthy controls in Chinese population (61). In the previous mentioned meta-analysis the results of 19 studies suggested no effect of rs1990760 polymorphism on GD either (57). That stays in agreement with our results and may suggest the possible association between previous viral infection and T1D but not ATIDs development and link the gap between environmental triggers and the development of the T1D in genetically predisposed patients.

It has been proposed that the rs231775 (A49G) polymorphism in exon 1 of CTLA-4 causes a the amino acid replacement (threonine to alanine) influencing the posttranslational modification of CTLA-4, resulting in decreased expression of CTLA-4 on the surface of T-cells. The presence of G allele thus leads to increased activation of T-cell, either autoreactive, causing the autoimmune response, clinically manifested for instance as AITDs or T1D (32, 62). In our study we observed a significantly dominant effect of G allele in rs231775 SNP in CTLA-4 gene in children with T1D in comparison to healthy controls and children with HT. There was also a significantly higher incidence of G/G an A/G genotype in patients with T1D. This might be suggestive of an increasing susceptibility to T1D of G allele in rs231775 SNP. Similar observations result from the recent meta-analysis of Chen et al. The analysis of 76 studies revealed that rs231775 polymorphism was associated with susceptibility to T1D in Caucasians and South Asians, moreover rs231775 polymorphism was also found to be significantly associated with susceptibility to type 2 diabetes (T2D) in East Asians and South Asians (62). The results of another meta-analysis stays in agreement with that indicating that G allele of rs231775 in CTLA-4 increases the risk of T1D development (18).

We did not reveal any statistical differences between any of AITDs children and controls in our study, thus according to our study CTLA-4 gene rs231775 polymorphism seems not to contribute to HT nor GD development in Polish children population. In accordance with our results the study of Narooie-Nejad et al. indicated no association of alleles and genotypes of the A49G in CTLA-4 polymorphism with HT in a group of patients from Iran (63). Similar conclusions come out of the cohort of Japanese and Brazilian patients (64, 65). However, in the study of Fathima et al. the association between rs231775 polymorphism of CTLA-4 gene was reported in patients with AITDs. The study revealed that G allele in rs231775 contributes to an increased incidence in HT and GD (15). Similarly in the study of Tu et al. in Chinese population G allele of rs231775 was revealed to be significantly more often in subjects with GD than in control subjects (66). Ting et al. found in their work that CTLA4 SNPs A49G was associated with GD not only in Chinese adults but also and children (67). In the study of Patel et al. the G allele, GG and AG genotypes in SNPs A49G were also more prevalent among autoimmune hypothyroidism patients in Indian population (68). Interestingly, a significant association with A49G SNP in CTLA4 appeared for GD and HT in a group of 64 pediatric Polish patients. There were also a significantly higher antithyroid antibodies titres associated with T allele in GD, and with G allele in HT patients observed (69).

Our study certainly has some limitations. First, the analysis in our work did not take into consideration the influence of environmental components apart from genetic factors on AITD and T1D susceptibility. We also acknowledge that we could not distinguish whether the associated alleles were causative factors or just markers linked with disease loci. Another limitation of the study is a relatively small sample size.

Genome-wide association (GWA) investigations indicate chromosome regions associated with particular autoimmune diseases. However, the localization of the most likely susceptibility locus requires numerous studies with genotyping in large samples. Here we demonstrated the contribution of polymorphic variants of genes encoding IL2RA, CTLA-4, and IFIH1 to autoimmune diseases predisposition.

In conclusion the rs7093069 T allele and T/T genotype was preponderant in children with T1D in comparison to healthy children as well as AITDs subjects, likewise rs1990760 T allele or T/T genotype had increased frequency in diabetic patients. Moreover, rs231775 G allele, A/G and G/G genotypes were also prevalent in T1D patients when compared with controls and HT. In contrast, there was no association found in rs7093069, rs1990760, and rs231775 polymorphisms with AITD susceptibility in our study. Thus, IL2RA polymorphism (rs7093069), IFIH1 polymorphism (rs1990760), and CTLA-4 rs231775) are associated with T1D susceptibility in Polish children and adolescents.

## Data Availability Statement

The raw data supporting the conclusions of this article will be made available by the authors, without undue reservation.

## Ethics Statement

The studies involving human participants were reviewed and approved by the Local Bioethical Committee at the Medical University of Bialystok. Written informed consent to participate in this study was provided by the participants' legal guardian/next of kin.

## Author Contributions

AB, AS, WM, and AK contributed conception and design of the study. NW-K organized the database. JG performed the statistical analysis. HB-S wrote the first draft of the manuscript. BS, BG-O, AK, and AŁ wrote sections of the manuscript. All authors contributed to manuscript revision, read and approved the submitted version.

## Conflict of Interest

The authors declare that the research was conducted in the absence of any commercial or financial relationships that could be construed as a potential conflict of interest.
